# Towards a Greater Understanding of Suicidal Behaviour and Its Prevention

**DOI:** 10.3390/ijerph15081629

**Published:** 2018-08-01

**Authors:** Merike Sisask, Kairi Kõlves

**Affiliations:** 1 Estonian-Swedish Mental Health and Suicidology Institute (ERSI), Tallinn 11615, Estonia; merike.sisask@tlu.ee; 2 School of Governance, Law and Society (SOGOLAS), Tallinn University, Tallinn 10120, Estonia; 3 Australian Institute for Suicide Research and Prevention (AISRAP), WHO Collaborating Centre for Research and Training in Suicide Prevention, Griffith University, Mt Gravatt Campus, Mount Gravatt QLD 4122 Australia

## 1. Introduction

Suicidal behaviour continues to be an important topic of research and significant public health concern globally [1,2,3]. Each year, approximately 800,000 people worldwide die by suicide, and by region, the rates are highest in Europe (especially Eastern Europe), South-East Asia, and in some regions of Africa [2,3,4]. There are notable gender and age differences in suicidal behaviour, and in most regions, males are associated with higher suicide rates than females and risk of dying by suicide is increasing with age [5]. Official suicide statistics are only the tip of the iceberg and the magnitude of the problem becomes evident when the whole range of suicidal behaviour—attempts, plans, thoughts—are taken into account [6].

The World Health Organization recommends a public health approach to prevent suicidal behaviour that focuses on identifying the patterns of suicide and suicidal behaviour of a group or population. The aim is to change environments in order to protect people against suicidality and to change the behaviour that put people at risk. The public health approach comprises surveillance, risk (protective) factor identification, prevention/intervention, and evaluation [3,7].

Research to date has shown that suicide prevention strategies with strong evidence are restricting access to lethal means, effective pharmacological and psychological treatments of depression, and school-based mental health awareness programmes. Other promising interventions, with insufficient evidence so far, are screening in primary care, primary care physicians’ education, general public education, media guidelines, gatekeeper training, and internet and helpline support [8,9,10].

A comprehensive meta-analysis of 50 years of suicide research literature, by Franklin et al. [11], identified a large number of risk and protective factors for suicidal thoughts and behaviour (suicide deaths and suicide attempts). However, they conclude that existing risk (and protective) factors are relatively weak and inaccurate predictors of suicidal thoughts and behaviour. Their findings indicate the need to understand that there are many different paths to suicidality and more risk factor studies, that must overcome the methodological limitations of the existing literature, are needed. Therefore, we must change our approach to conducting and evaluating studies.

The current special issue on advances in suicide research has brought together a collection of 25 articles from all over the world. Considering that suicidal behaviour is a multidimensional phenomenon, a variety of different topics are presented. It was a pleasure to note that the studies published in the special issue had utilised qualitative, quantitative, and mixed methods, which enrich our understanding of the contemporary world and enable conclusions to be drawn and deeper insight into phenomena—explanatory and explorative. Below is an overview that will present the main topics of the special issue on advances of suicide research, what value the published studies add to existing knowledge, and what remains for future research.

## 2. Peculiarities of Vulnerable Groups for Suicidality

Several articles targeted potentially vulnerable groups for suicidality (immigrants, Aboriginal people, LGB groups, youth living in slums, imprisoned persons) and indicated that vulnerability cannot be taken for granted and complexity within vulnerable groups should also be considered.

A study from Canada, a country primarily formed of immigrants and their descendants, investigated the “healthy immigrant effect” (immigrants are often found to be healthier both physically and mentally) and whether it exists for suicidal ideation [12]. The authors concluded that recent immigrants were less likely to report suicidal ideation than non-immigrants, but for established immigrants (living in Canada for 10 or more years), the risk of suicidal ideation was similar to non-immigrants. Furthermore, female immigrants had a much greater risk for suicidal ideation than males, irrespective of the duration of their stay in Canada, and were similar to non-immigrants.

The evidence shows that Aboriginal people, in comparison with the general population, have significantly higher suicide rates around the world, particularly in Australia and Canada. A study from Australia analysed the influence of coronial decision-making practices on the level of suicides among Indigenous Australians [13]. The authors claim that suicide rates are not simply an objective reflection of social truth and coronial determination is socially produced rather than existing as an objective reality. Thus, as Indigenous Australians are treated differently within the coronial system and the coroners anticipate a higher suicide rate among them, the coroners are a part of the mechanism of its production. The quality of suicide determination and registrations and its impact on official suicide statistics will remain an important subject in suicide research [14].

A study from Canada analysed protective factors among an Inuit population in Nunavut [15], as opposed to the majority of Aboriginal suicide research that has focused on risk factors. Protective factors were identified through content analysis of case vignettes and were divided into five groups: environmental, social, cultural, individual variables, and use of services. Beaudoin et al. [15] found that people without a history of suicide attempt had more protective factors throughout their lifespan than people who died by suicide and people who attempted suicide. Suicide attempters were more frequent health service users than the other two groups. Environmental protective factors, referring to stability and positive changes in the environment, showed the greatest difference between the three groups. The authors suggested that it is fair to believe that by implementing more protective factors early in the life cycle, it could lead to the development of more protective factors over the life course and reduce suicidal vulnerabilities.

A study among young French LGB adults demonstrated how the disclosure of sexual orientation as a stressful experience (one domain of the “minority stress”) is related to elevated suicide risk [16]. They explored associations between characteristics of stress (emotions, cognitive appraisal, and coping) and suicidal ideation and concluded that avoidance strategies had a direct effect on suicidal ideation, and mediated the link between primary appraisal (risk “Harm myself and others”) and suicidal ideation.

The findings of a study conducted among youth in the slums of Kampala, Uganda, underscored many unmet needs of this vulnerable population [17]. Nearly a quarter of study subjects had experienced suicidal ideation in the past year and suicidal ideation was associated with problem drinking and a range of adverse childhood experiences such as physical abuse, orphanhood, homelessness, and rape.

Another group vulnerable to suicidality are the imprisoned. Self-harm, which includes both suicidal and non-suicidal behaviour, is a common issue in detention globally. In the current special issue, two articles reported new findings about suicidality in detained persons. A study that was conducted in Switzerland investigated institutional factors, such as overcrowding and turnover, and discovered that along with already well-studied factors, these environmental factors play a crucial role—self-harm was higher when overcrowding and turnover increased, which also raises important human rights concerns [18]. Another study about risk factors in detention was conducted within the youth justice system in Sri Lanka, which helps to fill the gap in knowledge of detained young people from low- and middle-income countries [19]. This study identified high rates of self-harm, self-harm ideation, and self-harm with suicidal intent among these young people and several important psycho-social risk factors (e.g., being female, victimization of sexual abuse, exposure to self-harm by friends).

## 3. Gender Differences in Late Life Suicide Attempts

Although much research has been conducted about gender differences in suicidal behaviour, there is still limited knowledge about gender differences in late life suicide attempts. A Swedish study compared clinical characteristics of women and men aged 70 years and over that were hospitalised after a suicide attempt and found strikingly similar figures for depression, functional disability, and self-reported reasons for attempting suicide [20]. Only substance use disorder was a significantly different clinical variable and was noted more frequently in men.

## 4. Suicidality in the Family and Educational Setting Context

Three articles shed light on self-harm and suicidality in the family context. A systematic overview made an endeavour to identify measures to prevent the familial transmission of suicidal behaviour [21]. The authors found only a limited number of studies (only four articles, with three of them published on different aspects of the same study) about interventions that include the children of patients hospitalized with a suicide attempt. The lack of research in this area is striking, as this is a well-documented high-risk group of youth.

A different perspective of the family context was given in a systematic review by Curtis et al. [22]. Self-harm in youth is often stigmatised and misunderstood, which causes significant barriers to help-seeking for both parents and the youth themselves. The authors were interested in examining the perspectives of both young people (their expectations and preferences) and parents (their responses and feelings). This article highlights the need for accessible resources that seek to alleviate parents’ distress, influence the strategies implemented to manage the young person’s self-harm behaviour, reduce the self-blame of family members, and increase the likelihood of parental help-seeking [22].

An Australian study performed among the non-clinical adult population also highlighted the importance of family while talking about risk for self-injury [23]. The study examined the relationships between expressed emotions and shame, emotional involvement, depression, anxiety, stress, and non-suicidal self-injury in caregiving environments. In the final model, emotional involvement and overall shame were the only significant predictors of self-injury status. These findings have important treatment implications for engaging key family members in intervention and prevention efforts.

An article from Hungary about direct self-injurious behaviour compared two groups of youth—vocational school and high school students [24]. A vocational school is an educational setting generally associated with a lower socioeconomic status and this population is understudied. Direct self-injurious behaviour lifetime prevalence was significantly higher among the vocational students and it was associated with suicidal ideation. Students in the two groups also differed in the frequencies of certain life events.

## 5. Suicide Bereavement

The death of a close person by suicide is a severe trauma that can cause long-term negative consequences for both mental and physical health and increase the risk of suicide of those left behind. In the current special issue, three qualitative analyses are about suicide bereavement. Ross et al. [25] examined the individual experiences of both mothers and fathers bereaved by suicide in Australia. Qualitative analysis identified three key themes: searching for answers and sense-making, coping strategies and support, and finding meaning and purpose. The results indicated that adapting to bereavement is a dynamic and fluctuating process and there is a hope for parents that their traumatic loss may also present new pathways to personal growth, stronger relationships, and a greater appreciation for life.

Two analyses from the same study in the United Kingdom, about bereavement by suicide, were presented by Pitman et al. [26,27]. In the first paper, the authors explored the nature of young adults’ experiences of bereavement support and their suggestions regarding appropriate support provision [26]. The results showed that informal networks (family, friends, and peers) are the main source of emotional and practical support and even if offers are not taken up, knowing that potential support exists is comforting. Health professionals also have an important role and they are expected to be proactive. In the second paper, the objective was to further explore the impact of suicide on occupational functioning in work or educational settings [27]. It is already known that people bereaved by suicide have a greater probability to drop-out from work or school, but the current study adds the necessary context to it. The authors discovered specific aspects of grief—tearfulness, anger, reduced motivation, poor concentration, and anxiety—that have a major impact on educational and work performance. They also identified weaknesses in the institutional support and indications as to how these might be improved.

## 6. Telephone Crisis Helpline

The telephone crisis helpline that has a crucial role in suicide prevention was the topic of two articles from Australia [28,29]. In the first article, the authors explored whether the telephone crisis-line workers use patterns of signs to decide whether a caller might be suicidal, and whether these are influenced by caller characteristics such as gender [28]. Three patterns of suicide signs were uncovered—mood, hopelessness, and anger—which were different for male and female callers. The other paper specifically explored whether helpline callers’ gender is associated with telephone crisis-line workers’ ratings of callers’ suicide risk and their intention to use support-oriented or intervention-oriented skills [29]. The authors found that, generally, suicidal potential in both male and female callers expressing signs of suicide is recognized, but under some circumstances, the callers’ gender (being male) might influence telephone crisis-line workers’ intention to use intervention-oriented skills with the caller.

## 7. Copycat Effect and Contagion of Suicidal Behaviour

An important subject in suicide research is the copycat effect that is expressed in contagion of suicidal behaviour. This effect can be observed, for example, if suicide occurs in a school setting. The study by Gould et al. [30] conducted in the USA/New York State investigated the impact of a schoolmate’s suicide on the school’s student population overall. The study sought to determine whether there is excess psychological morbidity among students exposed to this event and whether students’ attitudes about coping and help-seeking strategies are more or less problematic. There was no excess of serious psychological morbidity (serious suicidality, depression) among the general population of students. However, two vulnerable groups were identified—students with recent negative life events and less close friends (but not the closest friends, as might be expected). Adaptive help-seeking attitudes increased in the general student population, but not for friends or for those with more negative life events.

Another study about the copycat effect investigated rather sensational and rare events—pilot-assisted suicides in the USA and Germany after the Germanwings 2015 incident in the French Alps [31]. Although rare events, they attracted significant media attention. The study results showed that in Germany, there were no aircraft-assisted pilot suicides and the relative aircraft-assisted pilot suicide risk for the USA was statistically non-significant. Responsible media coverage is important due to the large amount of publicity these events attract.

## 8. Help-Seeking in Military Context

Seeking help can be particularly complicated in special conditions, such as the military context, as service members believe seeking mental health treatment may negatively impact their military career. A study conducted among the U.S. Marine Corps tested if the service members who received care at military treatment facilities would be more likely to experience disruptions to their military careers [32]. Findings suggested that roughly half of treatment-seeking service members received potentially career-affecting treatment recommendations, and treatment-seeking service members were more likely to separate from military service, but no more likely to experience involuntary separation.

## 9. Aspects to Consider about Suicide Prevention in Clinical Context

Several articles reported the results from studies conducted in clinical settings. A study performed in Sweden about secondary prevention (i.e., targeted to high risk groups) tested the effectiveness of systematised mood-regulation focused cognitive behavioural therapy (MR-CBT) in comparison with the treatment as usual (TAU) [33]. At the end of treatment, the suicidal events were significantly reduced in the MR-CBT group, but not in the TAU group. Although the study sample was small, the results are encouraging enough to suggest that further studies should be undertaken.

The function of personality (as conceptualised by the Five-Factor model) in the Interpersonal-Psychological Theory of Suicide was explored in a Swiss clinical sample of suicidal patients [34]. Bearing in mind that other factors may play a more important role in suicidality than personality, the study results advocate personality as a potential determinant of the desire for suicide and the acquired capability for suicide as conceptualised by the Interpersonal-Psychological Theory of Suicide, a leading theoretical framework in suicidality. This provides health professionals with information that is potentially useful in their daily practice, for example, for constructing strong therapeutic alliances.

A study from Portugal aimed to describe health professionals’ practices when facing suicidal patients [35]. In general, they found it probable that health professionals (psychologists, psychiatrists, GPs) perform a comprehensive assessment of risk. A chain of care through a multidisciplinary approach was also scored as likely. The involvement of the family in the therapeutic process was considered with moderate probability. Formal instruments and protocols, including no-suicide contracts, are unlikely to be used. Training in suicide prevention and a higher number of patient suicide attempts were associated with a higher probability to perform a comprehensive assessment.

## 10. Economic Costs of Suicide

Although human suffering caused by suicidal behaviour is paramount, it would be useful to know what the economic costs of suicides for society are. An Australian study sought to estimate the economic cost of youth suicide (aged 15–24) [36]. The main outcome measure was the monetized burden of youth suicide and the costs were evaluated as direct costs, indirect costs, and intangible costs. Such information may be useful for informing policy in effective suicide prevention strategies.

## 11. Implications for Future Studies

Last but not least, an article from Australia highlights current status and reports on the next steps in suicide prevention research by analysing the published journal papers, funded grants, and views of stakeholders [37]. There has been strong emphasis on epidemiological studies, while funding for intervention studies has declined, despite the fact that stakeholders have always identified intervention studies as being the highest future research priority. Hence, as the authors declare, “If we are to make real advances in suicide prevention, we need to know what works, and identify and test effective interventions.”

There is a growing body of research in the field of suicide, this complex societal and yet individual phenomenon, and its prevention; there is a greater awareness that suicidal behaviour is preventable. This special issue has collected a broad range of high-quality papers that have made valuable contributions to the existing knowledge. Growing awareness of the vulnerability of diverse groups is informing better public health policy development. Tools such as social media provide new opportunities for raising awareness, detection, and prevention strategies. With these tools and a greater understanding of the socio-environmental factors influencing suicidal behaviour, more effective risk assessment and prevention strategies can be formed.

## References

[1] World Health Organization Global Health Observatory (GHO) Data. http://www.who.int/gho/mental_health/en/.

[2] World Health Organization (2018). Mental Health Atlas.

[3] World Health Organization (2014). Preventing Suicide: A Global Imperative.

[4] Värnik P. (2012). Suicide in the world. Int. J. Environ. Res. Public Health.

[5] Värnik P., Wasserman D., Kaschka W.P., Rujescu D. (2016). Global suicide. Biological Aspects of Suicidal Behavior.

[6] Bertolote J.M., Fleischmann A., De Leo D., Wasserman D., Wasserman D., Wasserman C. (2009). Suicidal thoughts, suicide plans and attempts in the general population on different continents. Oxford Textbook of Suicidology and Suicide Prevention: A Global Perspective.

[7] World Health Organization (2010). Towards Evidence—Based Suicide Prevention Programmes.

[8] Mann J.J., Apter A., Bertolote J., Beautrais A., Currier D., Mehlum L., Malone K., Marusic A., Lonnqvist J., Rutz W. (2005). Suicide prevention strategies: A systematic review. JAMA.

[9] Zalsman G., Hawton K., Wasserman D., van Heeringen K., Arensman E., Sarchiapone M., Carli V., Höschl C., Winkler P., Balazs J. (2017). Evidence-based national suicide prevention taskforce in Europe: A consensus position paper. Eur. Neuropsychopharmacol..

[10] Zalsman G., Hawton K., Wasserman D., van Heeringen K., Arensman E., Sarchiapone M., Carli V., Höschl C., Barzilay R., Balazs J. (2016). Suicide prevention strategies revisited: 10-year systematic review. Lancet Psychiatry.

[11] Franklin J.C., Ribeiro J.D., Fox K.R., Bentley K.H., Kleiman E.M., Huang X., Musacchio K.M., Jaroszewski A.C., Chang B.P., Nock M.K. (2017). Risk factors for suicidal thoughts and behaviors: A meta-analysis of 50 years of research. Psychol. Bull..

[12] Elamoshy R., Feng C. (2018). Suicidal ideation and healthy immigrant effect in the Canadian population: A cross-sectional population based study. Int. J. Environ. Res. Public Health.

[13] Tait G., Carpenter B., Jowett S. (2018). Coronial practice, indigeneity and suicide. Int. J. Environ. Res. Public Health.

[14] Värnik P., Sisask M., Värnik A., Laido Z., Meise U., Ibelshäuser A., Van Audenhove C., Reynders A., Kocalevent R.-D., Kopp M. (2010). Suicide registration in eight European countries: A qualitative analysis of procedures and practices. Forensic Sci. Int..

[15] Beaudoin V., Séguin M., Chawky N., Affleck W., Chachamovich E., Turecki G. (2018). Protective factors in the Inuit population of nunavut: A comparative study of people who died by suicide, people who attempted suicide, and people who never attempted suicide. Int. J. Environ. Res. Public Health.

[16] Charbonnier E., Dumas F., Chesterman A., Graziani P. (2018). Characteristics of stress and suicidal ideation in the disclosure of sexual orientation among young French LGB adults. Int. J. Environ. Res. Public Health.

[17] Culbreth R., Swahn M.H., Ndetei D., Ametewee L., Kasirye R. (2018). Suicidal ideation among youth living in the slums of Kampala, Uganda. Int. J. Environ. Res. Public Health.

[18] Baggio S., Gétaz L., Tran N.T., Peigné N., Chacowry Pala K., Golay D., Heller P., Bodenmann P., Wolff H. (2018). Association of overcrowding and turnover with self-harm in a Swiss pre-trial prison. Int. J. Environ. Res. Public Health.

[19] Hettiarachchi L.V., Kinner S.A., Tibble H., Borschmann R. (2018). Self-harm among young people detained in the youth justice system in Sri Lanka. Int. J. Environ. Res. Public Health.

[20] Wiktorsson S., Rydberg Sterner T., Mellqvist Fässberg M., Skoog I., Ingeborg Berg A., Duberstein P., Van Orden K., Waern M. (2018). Few sex differences in hospitalized suicide attempters aged 70 and above. Int. J. Environ. Res. Public Health.

[21] Lunde I., Myhre Reigstad M., Frisch Moe K., Grimholt T.K. (2018). Systematic literature review of attempted suicide and offspring. Int. J. Environ. Res. Public Health.

[22] Curtis S., Thorn P., McRoberts A., Hetrick S., Rice S., Robinson J. (2018). Caring for young people who self-harm: A review of perspectives from families and young people. Int. J. Environ. Res. Public Health.

[23] Hack J., Martin G. (2018). Expressed emotion, shame, and non-suicidal self-injury. Int. J. Environ. Res. Public Health.

[24] Horváth L.O., Balint M., Ferenczi-Dallos G., Farkas L., Gadoros J., Gyori D., Kereszteny A., Meszaros G., Szentivanyi D., Velo S. (2018). Direct self-injurious behavior (D-SIB) and life events among vocational school and high school students. Int. J. Environ. Res. Public Health.

[25] Ross V., Kõlves K., Kunde L., De Leo D. (2018). Parents’ experiences of suicide-bereavement: A qualitative study at 6 and 12 months after loss. Int. J. Environ. Res. Public Health.

[26] Pitman A., De Souza T., Khrisna Putri A., Stevenson F., King M., Osborn D., Morant N. (2018). Support needs and experiences of people bereaved by suicide: Qualitative findings from a cross-sectional British study of bereaved young adults. Int. J. Environ. Res. Public Health.

[27] Pitman A., Khrisna Putri A., De Souza T., Stevenson F., King M., Osborn D., Morant N. (2018). The impact of suicide bereavement on educational and occupational functioning: A qualitative study of 460 bereaved adults. Int. J. Environ. Res. Public Health.

[28] Hunt T., Wilson C., Caputi P., Wilson I., Woodward A. (2018). Patterns of signs that telephone crisis support workers associate with suicide risk in telephone crisis line callers. Int. J. Environ. Res. Public Health.

[29] Hunt T., Wilson C.J., Caputi P., Wilson I., Woodward A. (2018). The impact of caller gender on telephone crisis-helpline workers’ interpretation of suicidality in caller vignettes. Int. J. Environ. Res. Public Health.

[30] Gould M.S., Lake A.M., Kleinman M., Galfalvy H., Chowdhury S., Madnick A. (2018). Exposure to suicide in high schools: Impact on serious suicidal ideation/behavior, depression, maladaptive coping strategies, and attitudes toward help-seeking. Int. J. Environ. Res. Public Health.

[31] Laukkala T., Vuorio A., Bor R., Budowle B., Navathe P., Pukkala E., Sajantila A. (2018). Copycats in pilot aircraft-assisted suicides after the germanwings incident. Int. J. Environ. Res. Public Health.

[32] Ghahramanlou-Holloway M., LaCroix J.M., Koss K., Perera K.U., Rowan A., VanSickle M.R., Novak L.A., Trieu T.H. (2018). Outpatient mental health treatment utilization and military career impact in the United States marine corps. Int. J. Environ. Res. Public Health.

[33] Högberg G., Hällström T. (2018). Mood regulation focused CBT based on memory reconsolidation, reduced suicidal ideation and depression in youth in a randomised controlled study. Int. J. Environ. Res. Public Health.

[34] Baertschi M., Costanza A., Canuto A., Weber K. (2018). The function of personality in suicidal ideation from the perspective of the interpersonal-psychological theory of suicide. Int. J. Environ. Res. Public Health.

[35] Rothes I., Henriques M. (2018). Health professionals facing suicidal patients: What are their clinical practices?. Int. J. Environ. Res. Public Health.

[36] Kinchin I., Doran C.M. (2018). The cost of youth suicide in Australia. Int. J. Environ. Res. Public Health.

[37] Reifels L., Ftanou M., Krysinska K., Machlin A., Robinson J., Pirkis J. (2018). Research priorities in suicide prevention: Review of Australian research from 2010–2017 highlights continued need for intervention research. Int. J. Environ. Res. Public Health.

